# Female adolescents and the future of female genital mutilation/cutting: a report from an endemic area

**DOI:** 10.4314/ahs.v21i4.38

**Published:** 2021-12

**Authors:** Grace G Ezeoke, Abiodun S Adeniran, Kikelomo T Adesina, Adegboyega A Fawole, Munirdeen A Ijaiya, Adebunmi O Olarinoye

**Affiliations:** Department of Obstetrics & Gynecology, University of Ilorin/ University of Ilorin Teaching Hospital, Ilorin, Kwara State, Nigeria

**Keywords:** Female genital mutilation/cutting, female circumcision, harmful traditional practices, adverse childhood experiences

## Abstract

**Background:**

Despite collaborative efforts aimed at its eradication, Female Genital Mutilation/Cutting (FGM/C) continues in endemic areas.

**Objective:**

To evaluate the experience and preparedness of female adolescents to protect their future daughters from FGM/C.

**Methods:**

A cross-sectional survey involving adolescent secondary school girls in North Central Nigeria. Participants were secondary school students who completed the study's self-administered questionnaire after informed parental or participant's consent. Data management was with SPSS 20.0 (IBM, USA), P-value <0.05 was significant.

**Results:**

There were 2000 participants aged 13–19 years (mean 15.56±1.75), prevalence of FGM/C was 35.0%, awareness was 86.1%, mutilation was performed between infancy and eight years of age (mean 3.85±3.24 years), 644(32.2%) desire to mutilatetheir future daughters, 722(36.1%) expressed support for FGM/C and 63.1% of victims of FM/C reported adverse post-mutilation experiences. Support for FGM/C was associated with low social class (P0.0010), opinion that FGM/C has benefit (P0.001) and desire to mutilate future daughters (P0.001) while awareness of efforts to eradicate FMG/C was 813(40.7%).

**Conclusion:**

FGM/C remains prevalent with potential support for its continuation among female adolescents despite reported adverse post-mutilation experiences. The multi-pronged approach to eradicate FGM/C should prioritize re-orientation for adolescent girls, rehabilitation of mutilated girls and girl child formal education.

## Introduction

Female Genital Mutilation/ Cutting (FGM/C) is the removal of part or whole of the female external genitalia for non-medical reasons.^1^,^2^ It is an act of violence perhaps unintended against females with cultural and social coloration making it a mark of social integration in some communities.^1^ It was reported that over 66% of FGM/C occur in four countries i.e. Ethiopia (22%), Egypt (22%), Sudan (11%) and Nigeria (11%)2 despite regional, national and inter-national collaboration towards its eradication. Global statistics showed that the prevalence decreased between 2005 and 2010 at a 1% per year rate which if sustained will produce a 50% reduction by 2074. However, this appears inadequate; thus, an accelerated reduction has been suggested for efforts to ensure a 50% reduction by 2030 at an annual reduction rate of 3.2%^2^ although additional births will increase the number of vulnerable females during the same period. It was therefore projected that with the current prevalence, by 2030 about 20.7million girls aged 15–19 born between 2010 to 2015 may have been mutilated representing an additional increase. This is projected to represent an increase from 4.5million in 2010 to 7.8million by 2030 in West and Central Africa alone^2^ although the future risk for FGM/C varies depending on where the girl will live.

According to the report of the Multiple Indicator Cluster Survey (MICS) for 2016–17 by the National Bureau of Statistics in Nigeria,^3^ 18.4% of women aged 15–49 years had been mutilated, 61.8% were type I while the south-west had the highest regional prevalence of 41.1%. Among daughters aged 0–14 years, the national prevalence was 25.3%, the highest regional prevalence was in the North-west (56.0%) while 76.6% of the victims had type I mutilation. Awareness about FGM/C was 52.1% for women aged 15–49 years, 21.6% favoured its continuation while 76.5% supported its eradication.^3^

Efforts aimed at eradicating FGM/C include advocacy, educational activities to correct myths, emphasis on the possible medical complications as well as stimulation of dialogue among households, community groups, traditional, religious and political leaders. Older women who quite often encouraged FGM/C as well as men who are major decision makers in most endemic areas have been recruited into the fight against FGM/C while alternative sources of income were explored for the local circumcisers.^4^ Public figures and opinion leaders have accepted and functions as ‘champions’ to encourage others to discontinue the act, the need to empower and respect the rights of girls and women has taken centre stage while alternative harmless cultural rituals were introduced to replace FGM/C in some communities. A number of countries have passed laws to punish those involved in FGM/C while immigration laws in non-endemic countries have been broadened to accommodate asylum for those persecuted for FGM/C.^1^,^2^,^4^

It has been observed that while awareness about FGM/C continues to increase, this has not produced a commensurate behaviour change. Researchers have attempted the use of theoretical models of behaviour change to understand the impact of interventions to stop FGM/C on knowledge, beliefs, attitudes and behaviours of the people.^5^ For social and health issues, cognitive models have been developed to analyzeehaviour change for a variety of problems; these include health belief model, theory of planned behaviour and theory of reasoned action although diffusion theory and trans-theoretical model are the commonly applied.^6^ The diffusion theory focuses on the process by which an innovation is communicated through certain channels over time among members of a social system.^7^ The model recognizes that an individual or decision-making group undergoes a process as decision about innovation is not an instantaneous act; but a process that passes through the stages of knowledge, persuasion, decision, implementation and confirmation.^7^ The trans-theoretical or stage of change model focuses on integrating behaviour change theories used in psychology (especially for smoking cessation); the stages include pre-contemplation, contemplation, preparation, action, maintenance as well as termination.^8^ The theory has been extended to other behaviours such as substance abuse, dietary change, exercise promotion and safe sex.^9^ While none of these models is perfect for FGM/C, issues of individualism, self efficacy, negotiation and power/ influence are relevant. Combating FGM/C transcends the individual because individuals have to submit for scrutiny their perspectives based on experiences and influence in the social context of normality at family and community levels to effect a decision.^6^

The suggestion for a holistic approach to address all potential factors necessitated our effort to focus on adolescent girls who are potential future mothers and will have to make a decision whether to support the mutilation of their future daughters or otherwise. The study was aimed to determine the perception, experience and preparedness of adolescent girls to protect their future daughters from FGM/C.

## Methods

The study was a cross-sectional survey conducted among female adolescent secondary school students aged 13 to 19 years in eighteen secondary schools in North Central Nigeria. Participating schools were selected via multistage sampling from the list of all secondary schools in the study area with provision for equal number of public and private schools. After information about the study, informed consent was obtained from those aged 18years and above while parental consent was obtained for younger participants. The sampling method was purposive sampling; thereafter, each participant completed a self-administered questionnaire for the study. The inclusion criteria were female gender, studentship in any of the study sites at the time of the study, age 13 to 19 years and consent (parental or from the participant). Those outside the age bracket for the study or without consent for participation were excluded from the study. The lower limit of age 13 years was chosen because those younger had difficulty in completing the questionnaire during its pre-test.

The sample size was calculated using the formula^10^

n = 2 z^2^pq/d^2^

n= desired sample size

z= standard normal deviate set as 1.96 which corresponds to 95% confidence interval

p = proportion in the target population estimated to have FGM i.e. 0.253 (i.e. 25.3%).^3^

q = 1.0–0.253 = 0.747

d= degree of accuracy desired set at 0.05

n = 2 x1.96 x 1.96 x 0.253 x 0.747= 580.8 (0.05) ^2^

Thus, the minimum sample size was 581 + 116 (20% for non-respondents) = 697.

The information collected included demographic parameters, perception, attitude, experience about FGM/C as well as the attitude towards its eradication. Participants' confidentiality was maintained by using codes instead of individual names. Statistical analysis was with SPSS version 20.0 (IBM, USA), the Pearson's chi square was used for comparison with calculation of odds ratio at 95% confidence interval while P-value <0.05 was termed significant.

Ethical approvals were obtained from the ethical review committee of the teaching hospital as well as the Kwara State Ministry of Education and Human Capital Development before the commencement of the study. Participation was voluntary and recruitment was after obtaining an informed consent for participation. The authors declare no conflict of interest in the conduct of the study.

## Results

There were 2000 participants with mean age 15.56±1.75 (range 13–19) years, 121(6.1%) resided in rural area, 785(39.3%) were from low social class families, awareness about FGM/C was 86.1%, 1063(53.2%) have been aware for more than 4 years and the commonest source of information was the mother (1018; 50.9%) as shown in table 1.

**Table 1:** Socio-demographic characteristics of the participants

Socio-demographic variable	Frequency (N = 2000)	Percent
**Age**		
Range	13 – 19	
Mean age	15.56 ± 1.75	
**Residence**		
Rural	121	6.1
Urban	1879	94.0
**School location**		
Rural	157	7.9
Urban	1843	92.2
**Parents' social class**		
High	1215	60.8
Low	785	39.3
**Awareness about FGM**		
Yes	1721	86.1
No	279	14.0
**Duration of awareness about FGM/C (years)**		
<2	129	6.5
2–4	808	40.4
>4	1063	53.2
**Source of information** *		
Poster	29	1.5
Television	126	6.3
Books	135	6.8
Grandparents/ elders	138	6.9
Radio	150	7.5
Religious leaders	169	8.5
Father	389	19.5
Friends	473	23.7
Teacher	755	37.8
Mother	1018	50.9

* Multiple responses allowed

Table 2 shows that the prevalence of FGM/C among participants was 35.0% while 21.1% were unsure if they had been mutilated. The mean age at mutilation was 3.85±3.24 (range 1 to 8) years, 301(43.1%) described the experience as paiful, 140(20.0%) remembered that they bled excessively while 215(30.7%) could not remember the experience. Also, 669(33.5%) were aware that traditional circumcisers performs FGM/C while 41.6% opined that healthcare workers (28.3% for doctors and 13.3% for nurses) perform FGM/C. While 746(37.3%) opined that FGM/C has benefits, the common presumed benefits were control of sexual promiscuity (29.2%) and making a female complete (32.7%). In all, 958(47.9%) had seen a victim of FGM/C previously, the common victims were siblings (377; 39.3%), neighbor (307; 32.0%) or relatives (177(18.5) while 644(32.2%) reported the intention to circumcise their future daughters.

**Table 2:** Experiences and views about FGM/C among participants

Variables	Frequency (N = 2000)	Percent
**I have been mutilated**		
Yes	699	35.0
No	877	43.9
I don't know	424	21.1
**Mean age at mutilation (years)**	3.85±3.24 (range 1–8)	
**Description of mutilation experience (n=699)**		
Very painful	301	43.1
Excessive bleeding	140	20.0
I enjoyed it	43	6.2
I cannot remember	215	30.7
**In my opinion, FGM/C is performed by**		
Religious leaders	124	6.2
Nurse	265	13.3
Older women in the community	376	18.8
Doctor	566	28.3
Traditional circumciser	669	33.5
**Think that FGM/C has benefits**		
Yes	746	37.3
No	1254	62.7
**Presumed benefits of FGM/C (n=746)**		
Makes a woman complete	244	32.7
Controls sexual promiscuity	218	29.2
Removes bad part of female genitalia	149	20.0
Makes childbearing easier	135	18.1
**Seen a victim of FGM/C before**		
Yes	958	47.9
No	1042	52.1
**Description of the victim of FGM/C (n=958)**		
Playmate	137	14.3
Relative	177	18.4
Neighbor	307	32.0
Sister	377	39.3
**I will subject my future daughter to FGM/C**		
Yes	644	32.2
No	1356	67.8

Table 3 shows that awareness of efforts to eradicate FGM/C was 40.7% while the participants suggested community education and re-orientation (814; 40.7%) and announcements in the media (363; 18.2%). On seeing a girl that is to be mutilated, 643(32.2%) of participants intended to plead with the parents to desist from the act, 558(27.9%) intended to report to the police while 721(36.1%) described FGM/C as wickedness against women.

**Table 3:** Attitude and response to eradication of FGM/C

Variables	Frequency (N = 2000)	Percent
**Aware of efforts to eradicate FGM/C**		
Yes	813	40.7
No	1187	59.4
**Suggestion of activities to aid eradication of FGM/C**		
Community education/ re-orientation	814	40.7
Announcement on radio/television	363	18.2
Appeal to parents/adults	304	15.2
Arrest those assist in FGM/C	206	10.2
Punitive legislation against parents of the victims	313	15.7
**Possible response on seeing a girl about to be mutilated**		
Carry and hide the child	86	4.3
Take child to religious home	106	5.3
Stay and watch how it is performed	255	12.8
Take the child to the hospital	352	17.6
Report to the police	558	27.9
Beg the parents not to do it	643	32.2
**Description of FGM/C**		
Good for girls	355	17.8
Makes a girl to become a real woman	430	21.5
It is old-fashioned	494	24.7
Wickedness against women	721	36.1

Table 4 shows that majority of participants who were positively predisposed to FGM/C were from low social class families (P 0.001), resides in urban area (P0.001) and had seen a victim of FGM/C previously (P0.001). However, 16.8% of those who perceived FGM/C as wickedness against females and 38.0% of those thought it makes the women a real women planned to circumcise their children. Furthermore, significant number of girls who intended to mutilate future daughters supported the call to continue FGM/C (P0.001).

**Table 4:** Associated factors related to attitude concerning FGM/C

Variable	Should girls be circumcised			χ^2^	P-value
	Yes n (%)	No n (%)	I don't know n (%)		
**Social class**					
High	354 (49.0)	601 (73.4)	199 (43.4)	213.72	0.001
Low	368 (51.0)	218 (26.6)	260 (56.6)	42.46	0.001
**Residence**					
Rural	33 (4.6)	56 (6.8)	32 (7.0)	9.14	0.010
Urban	689 (95.4)	763 (93.2)	427 (93.0)	99.53	0.001
**School location**					
Rural	64 (8.9)	59 (7.2)	34 (7.4)	9.87	0.007
Urban	658 (91.1)	760 (92.8)	425 (92.6)	95.99	0.001
**Seen victim of FGM/C before**					
Yes	533 (73.8)	254 (31.0)	171 (37.3)	225.23	0.001
No	189 (26.2)	565 (69.0)	288 (62.7)	218.72	0.001
**Description of FGM/C?**					
Wickedness against females	121 (16.8)	477 (58.2)	123 (26.8)	349.59	0.001
Old fashioned	110 (15.2)	248 (30.3)	136 (29.6)	65.31	0.001
Good for girls	217 (30.1)	50 (6.1)	88 (19.2)	129.50	0.001
Makes someone a real woman	274 (38.0)	44 (5.4)	112 (24.4)	194.80	0.001
**FGM/C has benefits to girls**					
Yes	493 (68.3)	103 (12.6)	150 (32.7)	364.55	0.001
No	229 (31.7)	716 (87.4)	309 (67.3)	326.33	0.001
**Will mutilate future daughter**					
Yes	447 (61.9)	88 (10.7)	109 (23.7)	378.20	0.001
No	275 (38.1)	731 (89.3)	350 (76.3)	264.54	0.001
**Aware of effort to** **eradicate FGM/C**					
Yes	222 (30.7)	433 (52.9)	158 (34.4)	152.81	0.001
No	500 (69.3)	386 (47.1)	301 (65.6)	50.39	0.001

χ^2^: Chi square; *: Statistically significant (i.e. *p* value < 0.05)

Table 5 shows that the determinants of support for continuation of FGM/C were being a victim (OR0.191, 95%CI O.141–0.257; P0.001) or having the opinion that FGM/C is beneficial (OR0.108, 95%CI 0.080–0.145; P0.001) while seeing a victim of FGM/C (OR0.387, 95%CI 0.288–0.519; P0.001) encourages support for its eradication.

**Table 5:** Determinants of support to FGM/C among participants

Variable	P- value	OR	95% CI for OR	
			Lower	Upper
Awareness about FGM/C	0.517	0.858	0.540	1.363
Correct definition of FGM/C	0.636	0.928	0.680	1.266
A victim of FGM/C	0.001	0.191	0.141	0.257
Have seen a victim of FGM/C before	0.001	0.387	0.288	0.519
Opinion that FGM/C is beneficial to females	0.001	0.108	0.080	0.145

## Discussion

The prevalence of FGM/C among adolescent girls in the study was 35.0%; participants were mutilated at infancy to early childhood and about half could still recollect the trauma associated with the mutilation. A third of participants opined that FGM/C has some presumed benefits; therefore they expressed support for the act and intend to circumcise their future daughters. Also, about a third recalled having siblings who were victims of FGMC while less than half were aware of efforts aimed at its eradication. Determinants of support for eradication of FGM/C were awareness of efforts aimed at its eradication and participants who had seen a victim of FGM/C previously while those who presumed that FGM/C has some benefits or expressed desire to circumcise future daughters supported its continuation.

The prevalence of FGM/C in this study is higher than the national prevalence of 1 in 4 reported in the National Demographic Health Survey (NDHS)^11^ and 25.3% from the MICS in Nigeria.^3^ Compared to the MICS report, the 35.0% reported in this study is higher than the regional prevalence of 16.1% for north-central but comparable to the 40.6% reported for the state where the study was conducted.^3^ This suggests that the sample size in the study was representative of the situation in the state (locality); therefore, local reports may differ from national prevalence which is an aggregate value. However, the prevalence in this study is higher than 1.4% in Ghana and 14.6% in Kenya but lower than 80.7% in Egypt and 96.7% in Somalia.^12^ In a report from Ethiopia, the prevalence of FGM/C was 96% among adult women, 49% among their daughters less than 5 years and 91% in children less than one year.^13^ The differences may be a reflection of the variations in prevalence of FGM/C among countries.

FGM/C was performed before the eighth birthday in this study, this compares to reports of infancy from south-east Nigeria^14^ or age two years or less in Ethiopia;^13^ generally, mutilation was reported to be performed before age five in almost half of countries practicing it.^15^ FGM/C is performed mostly by local circumcisers as affirmed by majority (33.5%) of participants in this study; these are lay men and women who mutilate girls while the victims are physically restrained.^12^ This is associated with pain, apprehension and blood loss such that about two-thirds of participants in this study were able to recall such negative experiences of severe pain and bleeding after about a decade post-mutilation. Researchers have highlighted the impact of negative early life experiences on the health of individuals, such adverse childhood experiences (ACE) could be direct (abuse and neglect) similar to FGM/C or indirect through the living environments (parental conflict, substance abuse, mental illness).^17^ Reports indicated an association between ACEs and increased risk of poor health outcomes, drug addiction, interpersonal and self-directed violence^17^ as well as a consistent negative set of adult life outcomes.^18^ Although these were not evaluated in this study, efforts should be directed towards effective and widely available preventive interventions to counter these probable negative long-term sequelae of FGM/C as a form of ACE.

The high level of awareness about FGM/C in this study compares to 42.6%^19^ and 79.3%^20^ reported from studies in Nigeria; however, this has not changed the attitude to the act significantly. FGM/C has no health benefit; it causes physical and mental harm and is a human right abuse and breach of the basic right of girls and women by invading their healthy body tissue. Reported medical sequelae included labial adhesion and clitoral cyst,^21^ dysmenorrhea, obstructed labour and postpartum haemorrhage,^22^ wound infection, recurrent UTI,15 significantly higher risk for episiotomy, perineal tear, caesarean delivery and neonatal complications including stillbirth.^14^ Psychological sequelae and impaired sexual function can occur with all types of FGM/C,15 these may include loss of sexual desire, inadequate genital lubrication during coitus, inability to achieve orgasm and sexual dissatisfaction.^22^

However, despite the problems associated with FGM/C, the act persists; in a report from Nigeria, 33.3% of the victims expressed support for the act.^21^ A systematic review identified the factors favoring the perpetuation of FGM/C as an interplay of culture, tradition, sexual morals, marriageability, religion, presumed health benefits and male sexual enjoyment.^23^ In Nigeria, culture (60.8%),^14^ tradition (34.4%) and better marriage prospect (21.5%)^20^ have been identified as factors favouring continuation of FGM/C while this study identified the perception that FGM/C makes a woman complete, control of female sexuality and easier childbirth. Some culture regard FGM/C as a form of female gender identity aimed at preservation of family honour; therefore, many girls are forced to conform to avoid being teased or ostracised.^24^ The economic dimension of FGM/C encourages stitching of the introitus to guarantee virginity as a way to attract huge bride price in poor communities where the female child is considered a source of family income^24^ although this was not evaluated in this study.

In this study, 21% of respondents were unsure whether they have experienced FGM/C or not. Possible explanations for this include denial by mutilated individuals, lack of awareness due to less severe forms of FGM/C with minimal symptoms (type I), mutilation at very young age with failure of recall while some participants may not have been mutilated.^25^ This is an important limitation of studies based on self-reporting of FGM/C because confirmation by physical examination portends ethical challenges. In addition, certain discrepancies were noticed in the result of this study. This includes the observation that some participants who favoured continuation of FGM/C perceived the act as wickedness against women or indicated their unwillingness to circumcise their daughters. This underscores the dynamics of the non-linear relationship between information, intention and behavioural change. According to the trans-theoretical model, such individuals are reluctant abandoners because they have a high probability to abandon FGM/C despite their favour for its continuation provided other decision-makers or social pressure indicate otherwise.^9^ This group can be targeted for positive influence by ambassadors of FGM/C to discontinue the act. In addition, women who did not favour continuation of FGM/C and will not mutilate their daughters are called willing abandoners while those who supported the continuation of the act and plan to mutilate their daughters are termed willing adherents.^9^ Conversely, some women who supported the eradication of FGM/C may not act out their belief if the social context encourages reward for adherence or sanctions for non-conformance^25^, while 52.6% of mutilated women comprising mothers and grandmas insisted on female mutilation.^22^ A report among pregnant women in a rural north-west area in Nigeria indicated that 21.5% intended to mutilate the unborn children if they turned out to be daughters after delivery.^20^ In another study among school teachers, male teachers were the major initiator of their daughters' mutilation while grandmothers were the significant female proponent for FGM/C.^27^ Circumcisers derive remunerations from performing FGM/C while some depend solely on it for their livelihood. Therefore, they represent another important group of stakeholders who may encourage the continuation of the act.^16^

Moving forward in the effort to eradicate FGM/C, the multi-pronged approach remains relevant. The social and economic dimensions should be addressed because FGM/C is rooted in tradition and cultural beliefs which define the social values of any community. The communal force is such that members with dissenting opinion are forced to comply with the social norm to avoid embarrassment. In instances where dissenting members have acted out their disapproval, it has been met with significant communal outcry and reprimand. Therefore, since FGM/C thrives on the communal belief system, community re-orientation, education and innovation with alternative healthy ceremonies for initiation of young girls into adulthood has been suggested.^24^ In some communities, the birth of a female child is regarded as an investment which yield dividend as huge bride price. This is based on virginity at marriage prompting the use of FGM/C as a deterrent to premarital sex. Therefore, economic empowerment for the families in such areas should receive attention while the classification of FGM/C as a reportable health condition will be desirable. Furthermore, the inclusion of FGM/C and other gender-based violence in the school curriculum as a mode of dissemination of correct information to the students will improve their resilience to eradicate the act.

This study concludes that FGM/C remains prevalent with a probability that the female adolescent participants may support its continuation unless efforts at its eradication are prioritized. Also, FGM/C is a significant adverse childhood experience whose negative landmarks remained with its victims and portends a probable source of adolescent and adult psychological problems. The multi-pronged approach to eradicate FGM/C should prioritize re-orientation of adolescent girls, rehabilitation of mutilated girls and universal girl child formal education to equip the girls as advocates of the eradication of FGM/C.
